# Developmental competence of bovine oocytes selected based on follicle size and using the brilliant cresyl blue (BCB) test

**Published:** 2014-11

**Authors:** Hamed Karami Shabankareh, Golshan Azimi, Mehran Torki

**Affiliations:** *Department of Animal Sciences*, *Faculty of Agriculture, RaziUniversity, Kermanshah, Iran.*

**Keywords:** *Bovine*, *Oocyte*, *Development*

## Abstract

**Background:**

Many studies reported that follicle size has an essential role in developmental potential of oocytes. Also, the brilliant cresyl blue (BCB) test is one of the most important criteria in selection of more competent oocytes.

**Objective:**

Selection of developmentally competent bovine oocytes.

**Materials and Methods:**

A total of 1730 bovine cumulus oocyte complexes (COCs) were recovered from the ovaries by follicles isolation and classified into 3 categories according to the diameters of the follicles (small, <3 mm; medium 3-6 mm and large >6 mm). Oocytes were exposed to the BCB stain, diluted in Dulbecco's phosphate-buffered saline, modified with 0.4% bovine serum albumin (BSA) for 90 min. Oocytes with or without blue coloration of the cytoplasm were designated as BCB^+^ and BCB^-^, respectively.

**Results:**

The BCB^+^ and control oocytes originated from large and medium follicles exhibited a higher (p<0.0001) cleavage and blastocyst rate than BCB^-^ oocytes. Furthermore, the BCB^+^ oocytes from large and medium follicles had the highest (p<0.0001) proportion of blastocyst than other treatment groups. In contrast, the BCB^-^ oocytes from small follicles had the lowest (p<0.0001) proportion of blastocyst than other treatment groups. Interestingly, the percentage of the BCB^+^ oocytes from the large and medium ovarian follicles was significantly higher (p<0.0001), than the BCB^+^ oocytes from the small follicles.

**Conclusion:**

Current results confirmed that each BCB^+^ oocyte could not lead to perfect embryo development and the BCB test is not sufficient enough for the identification of oocytes that are competent for in vitro embryo development.

## Introduction

Mammalian immature oocytes are routinely selected for in vitro fertilization (IVF) on the basis of the visual assessment of morphological features, such as the thickness and compactness of the cumulus investment and the homogeneity of the ooplasm, this may reduce the yield of transferable embryos, as some oocytes with apparently normal morphology are in the early stages of degeneration (1). 

Consequently, morphological evaluation alone is insufficient to distinguish competent oocytes that have the ability to bring about full-term pregnancy (2). On the other hand, only 30-40% of the zygotes obtained after in vitro maturation and fertilization (IVF) will reach the blastocyst stage in culture (3). This is probably due to the quality of the oocytes at the beginning of maturation, and indicates the need for the development of other approaches. Different selection criteria have been used to predict the quality of these oocytes and to improve the embryo development such as oocyte diameter, brilliant cresyl blue (BCB) test and the follicle diameter (4-6). With the urgent need for establishing non-invasive option for embryo selection, the BCB test has been successfully used to differentiate oocytes (7-9).

The BCB stain is an electron acceptor, which can be used to semi-quantitate the level of glucose-6-phosphate dehydrogenize (G6PDH) activity in the oocytes, by modification of a visual color (10). G6PDH is known to be a component of the pentose phosphate pathway (PPP), which provides ribose phosphate for nucleotide synthesis and the formation of fatty acids. Moreover, the BCB test is based on the capability of G6PDH to convert the BCB stain from blue to colorless (active G6PDH: BCB^-^, inactive G6PDH: BCB^+^) (11). Therefore, the oocytes that have completed the growth phase are blue (BCB^+^) because the G6PDH activity is too low for stain reduction. Also, the growing oocytes become colorless (BCB^-^) due to G6PDH activity (12). Studies in small ruminants, pigs and heifer have shown that oocytes stained with BCB (BCB^+^) to be generally larger and more competent in maturation and developmental rate, than those unstained (BCB^-^) (5, 8, 11-13). 

Other authors have reported that the percentage of BCB^+^ oocytes developing to the morula and blastocyst stage were significantly higher than the control and BCB^-^ oocytes (8, 9). Also, follicle size has been the other important criterion used in selecting competent oocytes (14, 15). Likewise, the follicle size from which the oocytes are obtained characterizes the developmental stage of the follicle and the maturational stages of the oocyte within that follicle (16). Crozet *et al* described a positive relationship between follicular diameter, oocyte diameter, meiotic competence and embryonic development in goats (14). In other words, there is a relationship between follicle size and oocyte competence; the competence increases as the follicle enlarges (17). Oocytes from bovine follicles greater than 6 mm in diameter produce blastocysts in vitro at substantially greater rates than those from 2-6 mm follicles (17). 

Furthermore, follicles smaller than 2 mm yield oocytes capable of fertilization, but lack the ability to cleave beyond the 8-cell stage (15). Although, previous study evaluated the relationship between follicle size and oocyte selection using the BCB test, cleavage and blastocyst rate were not included (8). Due to the lack of reports about the effects of follicle size and BCB test in selection of developmentally competent bovine oocytes together following in vitro embryo production, therefore, the present study was conducted to use the BCB test as a selection criterion of developmentally competent bovine oocytes with every follicular origin (large, medium or small).

## Materials and methods

This prospective study was done in IVF and Embryo Transfer Laboratory at Razi University of Kermanshah, Iran (2011). The ethics committees of Razi University of Kermanshah approved this study.

**Chemicals**

All plastic ware used in the present experiments were obtained from Falcon, USA, while all chemicals and media were purchased from Sigma, USA. 

**Oocyte collection**

The number of 85 bovine ovaries were obtained from a local slaughterhouse and transported to the laboratory (within 2hr after slaughter) at 33-35^o^C in saline containing 50 IU/ml of penicillin and 50 µg/ml of streptomycin. Ovaries were washed three times in warm saline (8). The follicles were classified into 3 categories: small (<3 mm), medium (3-6 mm) and large (>6 mm) according to their diameters. The COCs from those follicles were aspirated using 21-guage needles attached to 10-mL syringe. 

A total of 1730 bovine COCs were recovered from different follicle diameters and used for the investigation. Of these, 640 were used for the production of control embryos. The medium used for recovery was TCM-199 with 25 mM HEPES supplemented with 50 IU/ml heparin, 50 µg/ml gentamicin and 4 mg/ml BSA. Only the oocytes with three or more complete layers of unexpanded cumulus cells and homogeneous cytoplasm were used.

**BCB test**

Immediately after collecting COCs from different follicle diameters, the number of 1090 COCs was washed three times in Dulbecco^’^s phosphate-buffered saline modified by the addition of 0.4% BSA (A-3311; mDPBS). Then the COCs were exposed to 26 µM of BCB (B-5388) diluted in mDPBS for 90 min at 38.5^o^C in humidified air atmosphere (8). After exposure to BCB, COCs from different follicle diameters were washed three times in mDPBS and classified into two groups, depending on their cytoplasm coloration: 616 oocytes with a blue cytoplasm (BCB^+^) and 474 oocytes without a blue cytoplasm (BCB^-^).

**In vitro maturation (IVM)**

The classified COCs were washed three times in the maturation medium. The maturation medium was TCM-199 supplemented with 0.23 mmol/L sodium pyruvate, 0.02 IU/ml porcine follicular stimulating hormone (p-FSH), 1 µg/ml 17β estradiol, 50 ng/ml epidermal growth factor (EGF), 10% (v/v) fetal calf serum (FCS) and 50 µg/ml gentamycin (8). COCs were placed in groups of 10-12 into 50 µl droplets of maturation medium under mineral oil. Maturation proceeded for 24 hr at 38.5^o^C in an environment of 5% CO_2_ in humidified air atmosphere.

**Sperm preparation**

Bovine spermatozoa were squeezed out from the caudal epididymis into 2 mL sperm tyrods albumin lactate pyruvate (TALP) medium (18). The motility of the sperm cells was evaluated under an inverted microscope and separated motile sperm fraction by swim-up. The top 1.5 ml of medium was then collected after incubation for 45 min at 38.5^o^C and 5% CO_2_. The pooled medium containing spermatozoa was washed twice (700 g for 5 min) with sperm TALP medium. The final pellet of spermatozoa was resuspended in the fertilization medium to a concentration of 20×10^6^ spermatozoa/ml. 

**IVF**

After IVM, The COCs were washed three times in the fertilization medium (TALP, 6) and transferred in groups of 10-12 to 45 µl of fertilization droplets. Insemination was carried out by adding 20×10^6^ spermatozoa/ml, 2 µg/ml heparin, and PHE (penicillamine, 20 µM; hypotaurin, 10 µM; epinephrine, 1 µM) (8). Oocytes were coincubated with spermatozoa for 22-24 hr at 38.5^o^C and 5% CO 2 in humidified air atmosphere.

**In** **vitro culture (IVC)**

Presumptive zygotes were denuded by pipetting using a small-bore pipette in synthetic oviductal fluid (SOF) +HEPES After washing, the zygotes were cultured in groups of 10-15 zygotes in 50 µl droplet of SOF modified by Takahashi and First at 38.5^o^C in a humidified atmosphere of 5% CO_2_, 5% O_2_ and 90% N_2_ (19). Cleavage was assessed after 48 hr of culture, and the numbers of embryos developing to the blastocyst stages were assessed on day 8. To prevent toxic accumulation of ammonium as a result of amino acid degradation, SOF medium was replaced every 48 hr. In this study, a two-culture system was used. The first system (SOF culture 1) medium for the first 48 hr, then, the medium was replaced by the second system (SOF culture 2) for the remaining 6 days of culture (20).

**Experimental design**

The developmental competence of the selected bovine oocytes by BCB test from different follicle diameters was evaluated. The follicles were aspirated at 33^o^C and divided according to their diameter into 3 groups- small follicles: <3 mm; medium follicles: 3-6 mm; and large follicles: >6 mm and the BCB test was used. The COCs were exposed to BCB staining (26 µM BCB in mDPBS) and oocytes were classified as BCB^+^ or BCB^-^ and without exposure to BCB (control). The cleavage and blastocyst rate were recorded for each follicle category. 

**Statistical analysis**

The experiment was conducted as complete randomized design. Data has been collected from nine experiment’s treatment in four replicates. Percentage values were logarithmic transformed and data analyzed by GLM procedure, followed by the LS means comparison by SAS statistical software (SAS Institute Inc., Cary, NC). Differences with a probability value of 0.05 or less was considered significant.

## Results

In table I the percentages of oocytes selected by BCB test is set out. Regardless of follicle diameters, there were no significant differences in proportion of BCB^+^ and BCB^-^ oocytes (56.34% and 43.66%, respectively). Table II shows the oocyte selection by the BCB test from different follicle diameters. The mean proportion of COCs classified as BCB^+^ in small, medium and large follicles were 45.14%, 64.56% and 59.30%, respectively (no significant difference between follicles group). The mean proportion of BCB^+^ oocytes (64.56%) of medium follicles was higher (p=0.0054) than those of the BCB^-^ oocytes (35.43 %). 

For large and small follicles, the percentage of oocytes classified as BCB^+^ and BCB^-^ was not significantly different (59.30%, 45.14% and 40.70%, 54.85%, respectively), but, in those BCB status, this difference was high numerically in large follicles. The percentages of cleavage and development to the blastocyst stage of oocytes selected by BCB from different follicles diameter has been shown in Table III. There were no significant differences between control and BCB^+^ oocytes in cleavage rate two days after IVF in all follicle diameters. 

Conversely, significant differences (p<0.0001) were recorded between BCB^+^ and BCB^-^ oocytes in cleavage rate in all follicle diameters. Furthermore, the cleavage rate was significantly low (p<0.0001) for BCB^-^ oocytes originated from all follicle diameters. After seven days of culture, BCB^+^ oocytes exhibited a higher (p<0.0001) blastocyst rate than BCB^-^ oocytes among all follicle diameters. Also, the percentage of blastocyst for BCB^+^ oocytes from large (26.65%) and medium (25.69%) follicles was respectively higher (p=0.0014, p=0.0022) than control (19.75%, 19.15%, respectively). There were no significant differences in blastocyst rate in BCB^+^ oocytes from large and medium follicles. 

The proportion of blastocyst from control and BCB^+^ oocytes originated from large and medium follicles were higher than (p<0.05) control and BCB^+^ oocytes originated from small follicles. 

There were no significant differences in blastocyst rate in BCB^-^ oocytes from large and medium follicles but significant differences were respectively (p=0.0190, p=0.0007) observed with BCB^-^ oocytes from small follicles (10.66%, 13.25% vs. 5.84%, respectively). In other way, the BCB^-^ oocytes originated from small follicles had the lowest (p<0.05) proportion of blastocyst than other treatment groups. In contrast, the BCB^+^ oocytes from large and medium follicles had the highest (p<0.05) proportion of blastocyst than other treatment groups. 

**Table I:** Oocyte selection by the BCB test

**Method of definition**	**Parameters**	
	**No. of oocytes**	**Each group %**
BCB^+^	616	56.34±6.32
BCB^-^	474	43.66±6.32

Note: Data expressed as mean ± SE

Mean Standard Error of Group: 0.08

BCB: Brilliant cresyl blue

BCB^+^: Oocytes with a blue cytoplasm

BCB^-^: Oocytes without a blue cytoplasm

**Table II:** Oocyte selection by the BCB test from different follicle diameters

**Follicle size**	**Method of definition**	**Parameters**		
		**No. of oocytes**	**Each group %**	**Total %**
Large	BCB^+^	175	59.30 ± 5.04^ab^	15.85 ± 1.65
	BCB^-^	138	40.70 ± 5.04^bc^	12.60 ± 1.22
Medium	BCB^+^	278	64.56 ± 5.46^a^	25.82 ± 2.51
	BCB^-^	158	35.43 ± 5.46^c^	14.33 ± 2.59
Small	BCB^+^	163	45.14 ± 8.47abc	14.84 ± 4.11
	BCB^-^	178	54.85 ± 8.47^ab^	16.54 ± 1.72

Note: Data expressed as mean ± SE.

Mean Standard Error of Group: 0.08

Mean Standard Error of Total: 0.10

BCB: Brilliant cresyl blue

BCB^+^: Oocytes with a blue cytoplasm

BCB^-^: Oocytes without a blue cytoplasm

a, b, c Different letters indicate statistical difference within each column (p<0.05).

**Table III:** Developmental competence of in vitro matured and in vitro fertilized oocytes selected by BCB from different follicle diameters

**Follicle size**	**Method of definition**		**Parameters**		
			**No. of oocytes**	**Percentage cleaved**	**Percentage blast** 1 **/ cleaved**
Large		Control	122	84.80 ± 1.06^a^	19.75 ± 0.98^b^
		BCB^+^	175	85.70 ± 1.50^a^	26.65 ± 1.51^a^
		BCB^-^	138	41.46 ± 0.50^b^	10.66 ± 2.12^d^
Medium		Control	353	82.43 ± 0.46^a^	19.15 ± 1.45^b^
		BCB^+^	278	83.51 ± 0.50^a^	25.69 ± 1.21^a^
		BCB^-^	158	42.45 ± 5.51^b^	13.25 ± 1.28^cd^
Small		Control	165	78.17 ± 3.68^a^	11.23 ± 1.91^cd^
		BCB^+^	163	82.93 ± 1.20^a^	14.76 ± 0.35c
		BCB^-^	178	39.31 ± 3.34^b^	5.84 ± 0.30^e^

Note: Data expressed as mean ± SE

Mean Standard Error of cleave: 0.01

Mean Standard Error of blast: 0.04

BCB: Brilliant cresyl blue

BCB^+^: Oocytes with a blue cytoplasm

BCB^-^: Oocytes without a blue cytoplasm

a, b, c, d, e Different letters indicate statistical difference within each column (p<0.05).

1 Blastocyst

**Figure 1:**
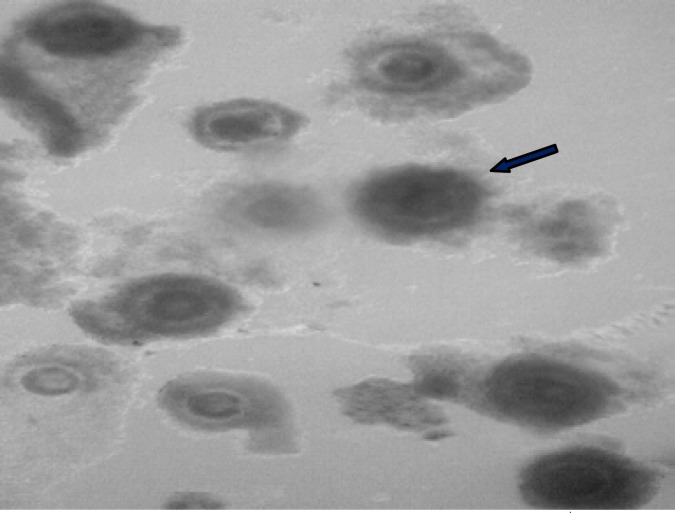
Immature bovine oocytes following BCB exposure. Arrow indicates BCB^+^ oocytes.

## Discussion

Studies conﬁrm that the BCB test is a good marker in pre-selection procedures of developmentally competent oocytes (8, 9). Regardless of follicle diameters, the percentage of BCB^+ ^oocytes (56.3%) obtained in the present study, employing 26 mM BCB, indicates that they had ﬁnished their growth phase and could be used for IVM/ IVF. This is in agreement with the result of Alm et al in cow oocytes, who observed that 57.9% of oocytes had a blue coloration after staining with BCB (21). Similar results reported in buffalo (53.9%); heifer (66%) (, ). 

Nevertheless, the percentage of BCB^+^ oocytes obtained in the present study was lower than percentages observed in pigs (81%) and cattle oocytes (70%) (, ). In contrast, in prepubertal goat oocytes the percentage of BCB^+^ and BCB^-^ oocytes was 30.1% and 69.9%, respectively (11). It is a hypothesis that these differences were caused by the selection criteria after BCB staining vary among laboratories. The current results show the difference in cleavage rate in BCB^+^ oocytes (range 85.70%-82.93%) in all follicle diameters and BCB^-^ oocytes (rang 41.46-39.31%) whereas these differences were significant. Similar ﬁndings were observed in other reports (23, 24). However, this is in contrast with the previous studies which found no difference in cleavage rate, based on BCB status (21, 25).

In the present study, BCB^+^ oocytes exhibited a higher blastocyst rate than BCB^-^ oocytes among all follicle diameters. These observations are in agreement with those of previous studies, in which the percentage of BCB^+^ oocytes developing to the blastocyst stage were significantly higher than BCB^-^ oocytes (7, 8, 26). These findings highlighting the ability of the BCB stain to be able to differentially select the developmentally competent oocytes. Despite higher blastocyst production with BCB^+^ oocytes than with BCB^-^ oocytes, no difference was found between BCB^+^ and control oocytes with bovine and equine oocytes (24, 27). 

Nevertheless, those ﬁndings are different from the other reports and the ones of the present study in large and medium follicles (5, 22). There is no clear reason for such discrepancy among studies. It may be associated with different morphological criteria used to select oocytes before exposing to BCB or control group. As current study demonstrates, there are no significant differences in blastocyst rate between BCB^+^ and control oocytes recovered from small follicles. Meanwhile, in the most studies, regardless of follicle diameters, those comparisons have been done and this could be another possible reason. Among ovarian populations, the oocytes that complete their growth show full competence for the resumption of meiosis and the completion of meiotic maturation. As a result of insufficient cytoplasmic maturation, BCB^-^ oocytes are not able to fully develop (28). 

The higher developmental competence of BCB^+^ oocytes, which are largely obtained from fully-grown follicles, can be referred to the better cytoplasmic maturation of these oocytes during the ﬁnal phases of folliculogenesis (24). Previous studies demonstrated that BCB^+^ oocytes contained higher number of mitochondrial DNA copies and had a greater diameter and larger cytoplasm volume in comparison with BCB^-^ oocytes (5). These factors have positive impact on fertilization and blastocyst rates (29).  Spikings *et al* reported that BCB^-^ oocytes are delayed in the onset of expression of proteins in comparison with BCB^+^ oocytes (30). Moreover, BCB^-^ oocytes had lower transcript level of genes involved in mitochondrial biosynthesis, suggesting that this may be one of the reasons for their low developmental competence compared to BCB^+^ and control oocytes (12). 

In the previous reports mentioned before, oocytes were obtained regardless of follicle diameters, resulting mostly, in the release of oocytes from small follicles (<2.0 mm), oocytes that are not competent for development. On the other hand, the present results demonstrated that the BCB^+^ oocytes from small follicles have lower blastocyst rate than BCB^+^ oocytes from medium and large follicles. Therefore, the developmental competence of oocytes is dependent on the size of follicle from which the oocyte is obtained (31). Moreover, the size of follicles is the most important criteria for oocyte selection (17). 

Various studies demonstrated that oocytes derived from large follicles are developmentally more competent than those derived from smaller follicles following IVF (6, 32). According to the results of the present study, oocytes isolated from 3-6 mm and >6 mm follicles consistently had a higher blastocyst rate than oocytes from <3 mm follicles in those BCB status (BCB^+^ and BCB^-^ oocytes). Also, the rate of blastocyst, in that BCB status following IVF was slightly, but not signiﬁcantly, greater for oocytes originating from large (26.65%) than those from medium follicles (25.69%). This was conﬁrmed in the other studies (17, 6). 

There is a positive relationship between follicle and oocyte diameter (32). Previous studies have indicated that oocytes from adult cows acquire developmental competence while the follicles grow from small to antrum size (33). The follicle must reach a diameter of at least 2-3 mm before the oocyte reaches a satisfactory developmental competence to blastocyst stage (34). It may be associated with that bovine oocytes from 2-mm follicles have not yet completed growth and RNA synthesis (34). 

The greater developmental competence of oocytes originating from large follicles (>6 mm) is probably due to differentiation that occurred at the more advanced stage of follicular development. There are several changes have been reported in large follicles like expression of LH receptors by granulosa cells, decrease of IGF-binding protein and increase of IGF-I within the follicular ﬂuid and increased expression of growth factors such as TGF-β, activin and inhibin. These happen simultaneously with ultrastructure changes within the oocyte and cumulus cells and with further growth of the oocyte (33). 

These may partly explain the better fertilization and development results observed for the oocytes from large and medium follicles in the present study. Based on the results each BCB^+^ oocyte could not lead to perfect embryo development and the BCB test is not sufficient enough for the identification of oocytes that are competent for in vitro embryo development.

## Note

***This article extracted from M.Sc. thesis. (Golshan Azimi)***
